# Association between locomotive syndrome and blood parameters in Japanese middle-aged and elderly individuals: a cross-sectional study

**DOI:** 10.1186/s12891-019-2480-9

**Published:** 2019-03-14

**Authors:** Toshinori Yoshihara, Hayao Ozaki, Takashi Nakagata, Toshiharu Natsume, Tomoharu Kitada, Yoshihiko Ishihara, Shuji Sawada, Masayoshi Ishibashi, Hiroyuki Kobayashi, Shuichi Machida, Hisashi Naito

**Affiliations:** 1 COI Project Center, Juntendo University, 2-1-1 Hongo, Bunkyo-ku, Tokyo, 113-8421 Japan; 2 School of Health and Sports Science, Juntendo University, 1-1 Hirakagakuendai, Inzai, Chiba 270-1695 Japan; 3 Graduate School of Health and Sports Science, Juntendo University, 1-1 Hirakagakuendai, Inzai, Chiba 270-1695 Japan; 4 Institute of Health and Sports Science & Medicine, Juntendo University, 1-1 Hirakagakuendai, Inzai, Chiba 270-1695 Japan; 5 Mito Medical Center, Tsukuba University Hospital, 1-1-1 Tennodai, Tsukuba, Ibaraki 310-0015 Japan

**Keywords:** Locomotive syndrome risk, Blood, HbA1c, Albumin, DHEA-S

## Abstract

**Background:**

Locomotive syndrome (LS) is associated with weakness and loss of function in the musculoskeletal organs. We aimed to determine the association between LS components and blood parameters in middle-aged and elderly individuals.

**Methods:**

We included 223 middle-aged and elderly individuals in this study (104 men and 119 women; age: 40–85 years). All participants were asked to fast for at least 3 h before the venous blood samples were obtained and the hemoglobin, total protein, glycated hemoglobin (HbA1c), growth hormone, albumin and lipid profile were measured. Three functional tests, the stand-up test, the two-step test, and the 25-question geriatric locomotive function scale (GLFS) were used to assess the risk of LS. Walking speed was assessed by the 10-m walking test. Maximal isometric muscle strengths of the knee extensors were examined, and the weight bearing index (knee extension strength/body weight) was calculated. To assess an independent association between blood parameters and LS, the area under the receiver operating characteristic curve analysis (area under the curve, sensitivity, and specificity) and a binary logistic regression analysis were performed with adjustment for age.

**Results:**

Of the 223 subjects, 119 (53.3%) fulfilled the diagnostic criteria for LS (including a two-step test score < 1.3, difficulty with one-leg standing from 40 cm in the stand-up test, and a 25-question GLFS score ≥ 7). Increased levels of HbA1c were significant risk factors for LS with an OR of 2.62 (OR_95%CI_ = 1.43–4.80), as determined by a logistic regression analysis. Additionally, dehydroepiandrosterone-sulfate (DHEA-S) levels were significant only in the male subjects (OR = 0.992 [OR_95%CI_ = 0.986–0.998]), at a threshold of 88 (AUC; 0.70, sensitivity; 79.6%, specificity; 49.1%). Moreover, 101 of 223 participants (41 men, 60 women) were analyzed for serum albumin levels, with a prevalence of LS at 55.4%, indicating that low levels of albumin were significant risk factors for LS (OR = 0.148 [OR_95%CI_ = 0.023–0.954], *p* = 0.0445).

**Conclusions:**

These results suggest that higher HbA1c and lower albumin are associated with the prevalence of LS in Japanese middle-aged and elderly individuals. Furthermore, low DHEA-S levels may be useful screening tools for LS in men.

## Background

Locomotive syndrome (LS) is the term proposed by the Japanese Orthopedic Association (JOA) to identify individuals with locomotive organ impairment [1]. LS leads to difficulties in the ability to stand, walk, run, climb stairs, and perform other physical functions essential to daily living and mobility [1]. This syndrome is caused by weakness and fragility of the function of musculoskeletal organs, such as bone, joint, and muscle. Any impairment in these organs results in pain, limited range of motion at the joints or spine, muscle weakness, and balance deficits, thereby resulting in limitations in the activities of daily living, reduction of quality of life, and necessity of nursing care [2]. Recently, LS evaluation methods have been established by the JOA, and include three functional tests: the stand-up test, two-step test, and 25-question geriatric locomotive function scale (GLFS) for assessing the risk of LS [3, 4].

Recent evidence has indicated that LS risk is ascertained by three risk indices that are based on age-dependent changes in healthy adults aged 23–95 years [1, 5]. Yoshimura et al. [1] examined the association between the LS risk test and a decline in mobility and demonstrated that three LS risk tests predict immobility and increased risk of LS. Moreover, Ogata et al. [5] determined whether these risk tests were beneficial as predictive screening programs for evaluating motor function and quality of life across a wide range of ages. Their findings suggest that this short battery of tests is a feasible tool for screening the adult population at risk of LS. Despite advances in screening methods of LS, the development of other predictive indicators of LS have not been established.

It is well known that blood parameters are effective screening tools for identifying the risk of metabolic syndrome and other multi-factorial diseases [6–8]. However, the association between blood parameters and LS has not been established in Japanese adults. Some investigators have demonstrated that dehydroepiandrosterone-sulfate (DHEA-S) has anti-aging effects [9], and circulating DHEA-S levels decline with age [10, 11]. Valenti et al. [12] revealed that low levels of DHEA-S are associated with muscle weakness in older men. Moreover, a decrease in the production of anabolic hormones, such as testosterone, growth hormone, and insulin-like growth factor-1 impairs the capacity of the skeletal muscles to incorporate amino acids and synthesize proteins [13]; therefore, these parameters were thought to be associated with the prevalence of LS. However, the association between these blood parameters and the components of LS remains unclear. Therefore, the purpose of this study was to determine the association between the components of LS and blood parameters in Japanese middle-aged and elderly people.

## Methods

### Subjects and measurements

We performed a cross-sectional analysis between May 2015 and November 2016. In total, 223 untrained community-dwelling middle-aged and elderly individuals (104 men and 119 women; ages: 40–85 years; mean height: 159.3 ± 8.2 cm; mean weight: 58.4 ± 9.5 kg) were included in this study. The participants had not performed any resistance training for at least 1 year prior to the start of the study. We excluded individuals who were unable to follow our instructions and those with chronic orthopedic conditions or any health or medical condition that limited the ability to undertake light-to-moderate walking. In addition, the participants completed a self-report questionnaire regarding medical history and comorbid conditions. All participants were informed about the contents of the study, and they provided a signed informed consent. This study was approved by the Ethics Committee of the Juntendo University (Approval Number: 27–10).

Body weight, skeletal muscle mass, fat mass, and percentage of body fat (% Fat) were measured via bioelectrical impedance analysis using a body composition analyzer (InBody730; Biospace). Venous blood samples of about 13 mL were obtained between 10:00 am and 5:00 pm after at least a 3-h fast, and the following parameters were analyzed: white blood cell (WBC) count, hematocrit (Hct) value, and levels of hemoglobin (Hb), total protein (TP), triglyceride (TG), low density lipoprotein (LDL)-cholesterol (C), high density lipoprotein (HDL)-C, HDL/LDL-C ratio, glycated hemoglobin (HbA1c) (National Glycohemoglobin Standardization Program [NGSP]), aspartate amino transferase (AST/GOT), alanine amino transferase (ALT/GPT), alkaline phosphatase (ALP), leucine aminopeptidase (LAP), γ-glutamyl transpeptidase (γ-GTP), growth hormone (GH), insulin-like growth factor-1 (IGF-1), testosterone, cortisol, dehydroepiandrosterone-sulfate (DHEA-S), and albumin. Of 223 participants, 101 (41 men, 60 women) were analyzed for albumin levels because we adopted it as an indicator after October 2015. All assays were performed by SRL Inc. (Tokyo, Japan).

After blood collection, all participants were measured for LS risk, walking speed, and maximal isometric muscle strength. LS risk tests (the stand-up test, two-step test, and 25-question GLFS) were performed as described previously [14]. Briefly, in the stand-up test, the participants were asked to stand from a sitting position on two legs or one leg, and from four different seat heights (40, 30, 20, and 10 cm). Participants were instructed to stand up without leaning back to gain momentum and to maintain the standing posture for at least 3 s. A score from “0” to “8” was allocated based on the difficulty, as described by Ogata et al. [5]. For the two-step test, participants stood with the toes of both feet behind the starting line and performed two maximal stride lengths, one with each foot, and the distance from the starting line was measured. The score was calculated as the length of the two steps (cm)/height (cm) [5]. The 25-question GLFS is a self-reported questionnaire, which is a comprehensive measure consisting of 25 items, including pain, activities of daily living, and mental health during the last month [3]. These 25 items were graded on a five-point scale, from 0 (no impairment) to 4 (severe impairment) points, with the scores summed to provide the total GLFS score; a higher GLFS score was associated with a higher risk of developing LS. Our previous study indicated that the measured variables from the stand-up test, two-step test, and GLFS have enough validity and reliability, with the intra-class correlation coefficients being 0.87, 0.93, and 0.76 and Cronbach’s α being 0.93, 0.95, and 0.88, respectively [14].

Walking speed was evaluated by timing each subject as they walked across a 10-m corridor on a hard-surfaced floor. They were asked to walk down the corridor as fast as possible without running. The maximum isometric strength of the knee extension was also measured (Takei, Tokyo, Japan) as described previously [14]. Each subject was seated on a chair with the hip joint angle at 90° flexion (0° = full hip extension), and they were instructed to perform maximum isometric knee extensions two or three times. The best recorded value was used as the representative one, and the weight bearing index (knee strength/body weight) was calculated.

### Definition of locomotive syndrome

According to the results of the LS risk test, the participants were classified as having LS when a participant met one or more of the following criteria: (1) difficulty in standing from a seat at a height of 40 cm using one leg in the stand-up test (either leg), (2) two-step test score < 1.3, and (3) GLFS score ≥ 7 [15]. All other participants were placed in the non-LS group. In this study, we focused on the stage 1 of JOA definition of LS considered as LS group (including stage 1 and 2), and the independent values were compared between the LS and non-LS groups.

### Statistical analysis

The data are presented as the mean ± standard deviation (SD). To compare the variables between the LS and non-LS groups, we used Student’s unpaired *t*-test. For variables that did not show normal distribution (for HbA1c levels and DHEA-S levels in males), the Mann-Whitney U test was used. The medians [interquartile ranges (IQRs)] are also presented for variables that did not show a normal distribution (i.e., for HbA1c and DHEA-S levels in males). The Benjamini-Hochberg procedure was performed to control the false discovery rate at 0.05 (used 14 tests for blood characteristics and biochemical examinations and 6 tests for hormone levels in males and females, respectively) [16]. To determine whether blood parameters were associated with LS risk, a binary logistic regression analysis (adjusted for age) was performed for HbA1c, DHEA-S levels in males, and albumin. We used the odds ratio (OR) and 95% confidence intervals (OR_95%CI_) to estimate the relative risk. A receiver operating characteristics (ROC) curve was used to select an appropriate cutoff and to determine the area under curve (AUC) and maximum sensitivity and specificity. Correlations between HbA1c and albumin levels and characteristics of participants [% Fat, body mass index (BMI), skeletal muscle mass, 10-m walking speed, and the weight bearing indices of knee extension] were analyzed using simple linear regression and Pearson’s correlation analysis. All of the statistical analyses were performed using the EZR package (Saitama Medical Center, Jichi Medical University) [17], which is a modified version of the R programming environment (The R Foundation for Statistical Computing, Vienna, Austria). The statistical significance level was set at *p* <  0.05.

## Results

### Characteristics of subjects

Table 1 shows the characteristics of the participants. In this study, 53.3% (119 of 223 participants: 55 men, 64 women) of participants fulfilled the diagnostic criteria for LS. Age, weight, and body fat percentage in the LS group were significantly higher than those in the non-LS (control) group (Table 1, *p* <  0.05). There was no significant difference between the non-LS and LS groups with respect to height, BMI, and total skeletal muscle mass (Table 1). The maximal isometric muscle strength of knee extension and the weight bearing indices of knee extension were significantly lower in the LS group than in the control group. Additionally, the 10-m walking speed was significantly lower in the LS group than in the control group. The stand-up and two-step test scores in the LS group were significantly lower than those in the non-LS group (3.95 ± 1.05 vs. 5.25 ± 0.57 and 1.34 ± 0.11 vs. 1.48 ± 0.10, respectively). In addition, the GLFS scores in the LS group were significantly higher than those in the non-LS group (6.03 ± 4.63 vs. 2.00 ± 1.70).

**Table 1:** Characteristics of participants without locomotive syndrome (non-LS) and those with locomotive syndrome (LS)

	non-LS			LS			*p value (non-LS* vs *LS)*
	All (*n* = 104)	male (*n* = 49)	female (*n* = 55)	All (*n* = 119)	male (n = 55)	female (*n* = 64)	
Age (years)	66.4 ± 7.3	68.7 ± 6.2	64.3 ± 7.6	70.0 ± 6.4	71.8 ± 6.3	68.5 ± 6.1	0.0001*
Height (cm)	158.4 ± 7.3	164.2 ± 4.9	153.2 ± 4.6	160.2 ± 8.8	166.2 ± 6.7	155.0 ± 6.9	0.0931
Weight (kg)	56.9 ± 8.9	61.8 ± 7.3	52.5 ± 7.9	59.7 ± 9.8	64.9 ± 8.0	55.3 ± 9.0	0.0245*
Body fat percentage (%)	26.1 ± 7.6	21.4 ± 5.7	30.3 ± 6.6	28.5 ± 7.8	24.1 ± 5.7	32.3 ± 7.3	0.0212*
BMI (kg/m^2^)	22.6 ± 3.0	22.9 ± 2.4	22.4 ± 3.3	23.0 ± 4.5	23.5 ± 2.4	23.0 ± 3.4	0.1375
Total skeletal muscle mass (kg)	22.8 ± 4.3	26.6 ± 2.3	19.3 ± 2.1	23.0 ± 4.5	26.9 ± 2.9	19.6 ± 2.5	0.7151
10-m walking (m/s)	2.1 ± 0.3	2.2 ± 0.4	2.0 ± 0.2	1.9 ± 0.3	2.0 ± 0.4	1.8 ± 0.2	< 0.0001*
KE WBI (kg/kg Weight)	0.77 ± 0.17	0.81 ± 0.14	0.73 ± 0.19	0.66 ± 0.16	0.72 ± 0.15	0.60 ± 0.14	< 0.0001*
Stand-up test (score)	5.25 ± 0.57	5.22 ± 0.55	5.27 ± 0.59	3.95 ± 1.05	3.84 ± 1.10	4.05 ± 1.00	< 0.0001*
Two-step test (score)	1.48 ± 0.10	1.48 ± 0.11	1.48 ± 0.10	1.34 ± 0.11	1.35 ± 0.12	1.32 ± 0.11	< 0.0001*
25-question GLFS (score)	2.00 ± 1.70	1.90 ± 1.70	2.09 ± 1.71	6.03 ± 4.63	5.40 ± 5.08	6.56 ± 4.18	< 0.0001*

Values are means ± standard division (SD). *LS* locomotive syndrome; *non-LS* without locomotive syndrome; *BMI* body mass index, *KE* knee extension, *WBI* weight bearing index; *GLFS* Geriatric Locomotive Function Scale.* *p* < 0.05

### Blood characteristics and biochemical examinations

The blood parameters of the LS and non-LS groups are shown in Table 2 with adjusted *p* values. The LS group had higher HbA1c levels than the non-LS group (*p* <  0.05). However, no significant differences were observed in terms of WBC count, HDL/LDL-C ratio, and Hb, Hct, TP, TG, AST, ALT, ALP, LAP, and γ-GTP levels in both groups (*p* > 0.05).

**Table 2:** Comparison of blood parameters between participants without locomotive syndrome (non-LS) and those with locomotive syndrome (LS)

	non-LS (*n* = 104)	LS (*n* = 119)	*Benjamini-Hochberg p value*
WBC (cells/μL)	5391 ± 1179	5734 ± 1759	0.4349
Hb (g/dL)	13.8 ± 1.2	13.6 ± 1.4	0.6161
Hct (%)	41.5 ± 3.2	41.0 ± 3.5	0.7084
TP (g/dL)	7.30 ± 0.40	7.25 ± 0.38	0.6389
TG (mg/dL)	132.6 ± 66.9	148.0 ± 89.3	0.5282
LDL-C (mg/dL)	119.5 ± 27.8	119.3 ± 29.8	0.9431
HDL-C (mg/dL)	67.5 ± 16.7	65.2 ± 17.8	0.6370
LDL/HDL	1.88 ± 0.62	1.98 ± 0.72	0.7792
HbA1c (%) NGSP	5.48 ± 0.44 5.50 (5.20–5.70)	5.75 ± 0.58 5.70 (5.35–6.00)	0.0028*
AST/GOT (U/L)	24.9 ± 6.9	25.6 ± 8.6	0.5955
ALT/GPT (U/L)	22.0 ± 10.4	23.0 ± 12.6	0.5908
ALP (U/L)	206.2 ± 61.1	226.6 ± 64.9	0.1190
LAP (U/L)	52.4 ± 9.5	53.3 ± 9.5	0.5719
γ-GTP (U/L)	30.6 ± 26.1	34.4 ± 36.5	0.5816

Values are expressed as means ± standard division (SD). The medians [interquartile ranges (IQRs)] are also presented for variables that did not show a normal distribution (for HbA1c). *WBC* white blood cell, *Hb* hemoglobin, *Hct* Hematocrit, *TP* Total protein, *TG* Triglyceride, *LDL-C* low density lipoprotein cholesterol, *HDL-C* high density lipoprotein cholesterol, *NGSP* National Glycohemoglobin Standardization Program, *AST/GOT* aspartate amino transferase, *ALT/GPT* alanine amino transferase, *ALP* alkaline phosphatase, *LAP* leucine aminopeptidase, *γ-GTP* γ-glutamyl transpeptidase. * *p* < 0.05

An ROC analysis was conducted for each blood parameter, and the threshold for discriminating the non-LS and LS groups was identified, which was 5.7% for HbA1c (AUC; 0.64, sensitivity; 70.2%, specificity; 54.6%). A higher HbA1c level was a significant risk factor for LS, with an OR of 2.62 (OR_95%CI_ = 1.43–4.80), as determined by the logistic regression analysis (*p* = 0.0018).

The linear regression analysis revealed significant positive or negative correlations between HbA1c level and BMI or 10-m walking speed (BMI, r = 0.1628; 10-walking speed, r = − 0.2206, *p* <  0.05) (Table 3). In addition, significant differences were present in HbA1c level between LS and non-LS when the two functional tests (stand-up test or two-step test) were used for classification (*p* = 0.0008 or *p* = 0.132) (Table 4).

**Table 3:** Correlation between the characteristics of participants and blood parameters

	Body fat percentage (%)	BMI (kg/m^2^)	Total skeletal muscle mass (kg)	10-m walking (m/s)	KE WBI (kg/kg Weight)
HbA1c (%) NGSP *n* = 223 (104, 119)	0.1358	0.1628*	0.0590	−0.2206*	−0.1332
Albumin (g/dL) *n* = 101 (41, 60)	0.2149*	0.1391	−0.2196*	−0.0385	0.0872

Values are means ± standard division (SD). *NGSP* National Glycohemoglobin Standardization, *BMI* body mass index, *KE* knee extension, *WBI* weight bearing index. n (male, female). * *p* < 0.05

**Table 4:** The differences among locomotive syndrome (LS) classifications by three different LS risk tests and comparison of blood parameters between participants without LS (non-LS) and those with LS

	n (male, female) non-LS: LS	non-LS	LS	*p value*
HbA1c (%) NGSP				
Stand-up test	140 (61,79): 83 (43,40)	5.53 ± 0.46	5.79 ± 0.61	0.0008*
Two-step test	180 (86,94): 43 (18,25)	5.58 ± 0.50	5.81 ± 0.65	0.0132*
25-question GLFS	167 (84,83): 56 (20,36)	5.59 ± 0.48	5.74 ± 0.67	0.0756
Albumin (g/dL)				
Stand-up test	60 (19,41): 41 (22,19)	4.37 ± 0.27	4.26 ± 0.22	0.0890
Two-step test	81 (34,47): 20 (7,13)	4.35 ± 0.25	4.21 ± 0.22	0.0178*
25-question GLFS	74 (34,40): 27 (7,20)	4.34 ± 0.27	4.27 ± 0.21	0.1950

Values are means ± standard division (SD). *NGSP* National Glycohemoglobin Standardization, *GLFS* Geriatric Locomotive Function Scale. * *p* < 0.05

### Hormone levels

**Fig. 1:**
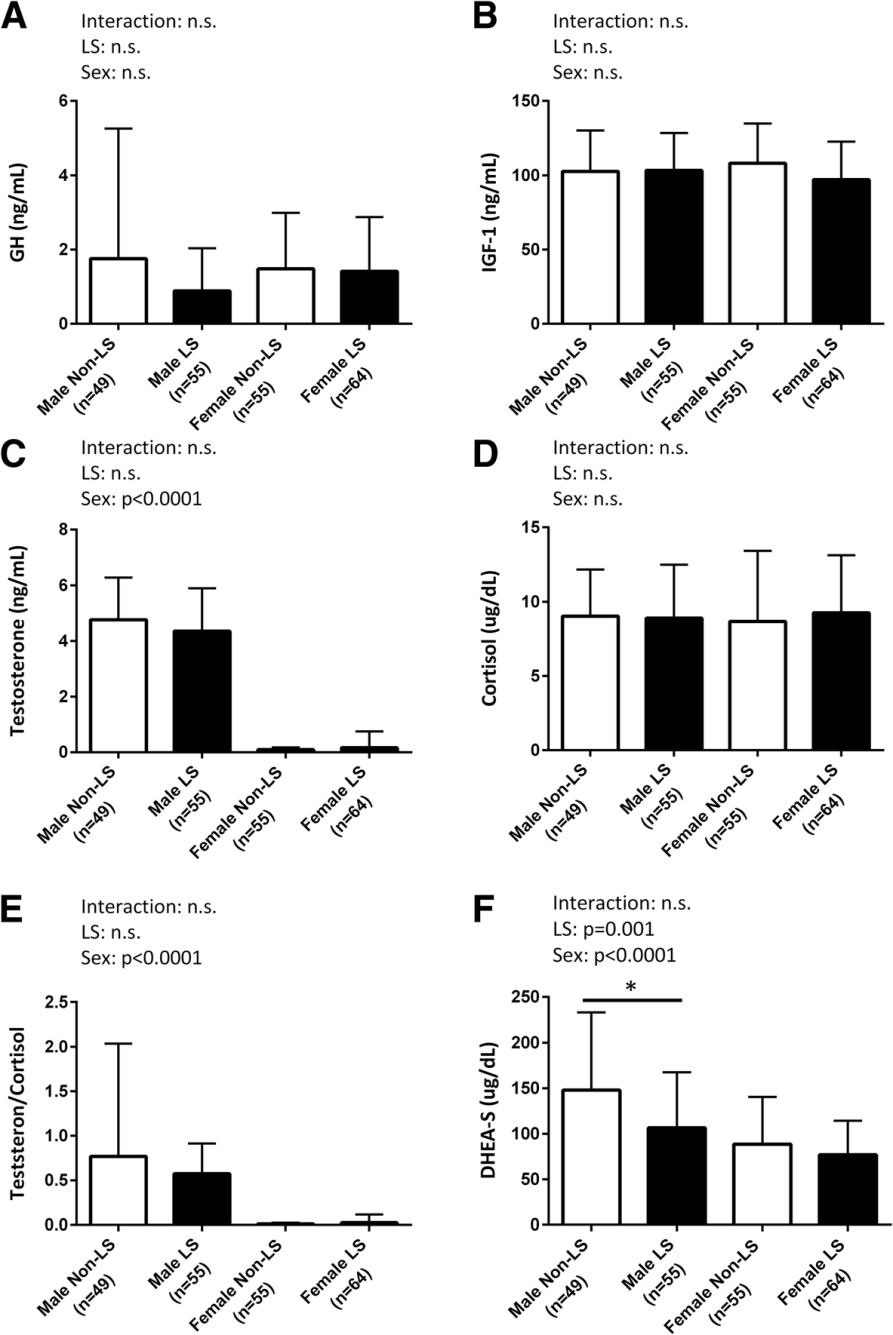
Comparison of blood hormone concentrations between non-locomotive syndrome and locomotive syndrome in male and female participants. **a**) GH, **b**) IGF-1, **c**) Testosterone, **d**) Cortisol, **e**) Testosterone/Cortisol ratio, and **f**) DHEA-S levels. Values are means ± standard division (SD). The medians [interquartile ranges (IQRs)] are presented for DHEA-S levels in males, which did not show normal distribution: 119.0 (94.0–174.5) for non-LS and 93.0 (61.0–150.8) for LS. GH: growth hormone, IGF-1: insulin-like growth factor-1, DHEA-S: dehydroepiandrosterone-sulfate. □:Non-LS participant, ■: LS participant. * *p* < 0.05 vs. each gender. The results of two-way ANOVA are displayed

Figure 1(A-F) shows the comparison of blood hormone concentrations between the non-LS and LS groups in the male and female participants. The two-way ANOVA revealed that there was an association between the prevalence of LS and DHEA-S level, and the male participants had significantly higher DHEA-S levels than the female participants (Fig. 1F). In addition, DHEA-S levels in male participants were significantly lower in the LS group than in the non-LS group (men in non-LS: 148.1 ± 85.5; men in LS: 106.6 ± 61.0; women in non-LS group: 88.7 ± 51.8; women in LS group; 77.1 ± 37.5 μg/dL). The levels of other hormones did not present statistically significant difference regardless of LS risk in both sexes.

In the male subjects, the ROC analysis indicated that the cutoff point was 88 μg/dL (AUC; 0.70, sensitivity; 80.2%, specificity; 59.8%), and low level of DHEA-S was a significant risk factor for LS (*p* = 0.0088) with an OR of 0.992 (OR_95%CI_ = 0.986–0.998).

### Albumin level

**Fig. 2:**
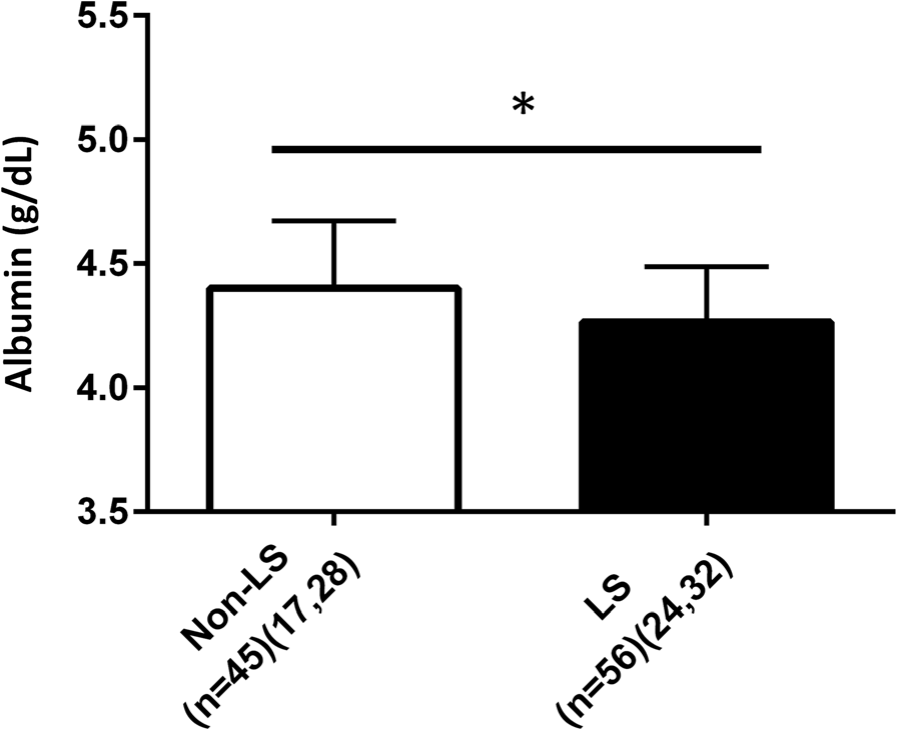
Serum albumin levels between participants without locomotive syndrome and those with locomotive syndrome [n (male, female) = Non-LS: 45 (17, 28); LS: 56 (24, 32)]. Values are means ± standard division (SD). □: Non-LS participant, ■: LS participant. * *p* < 0.05

**Fig. 3:**
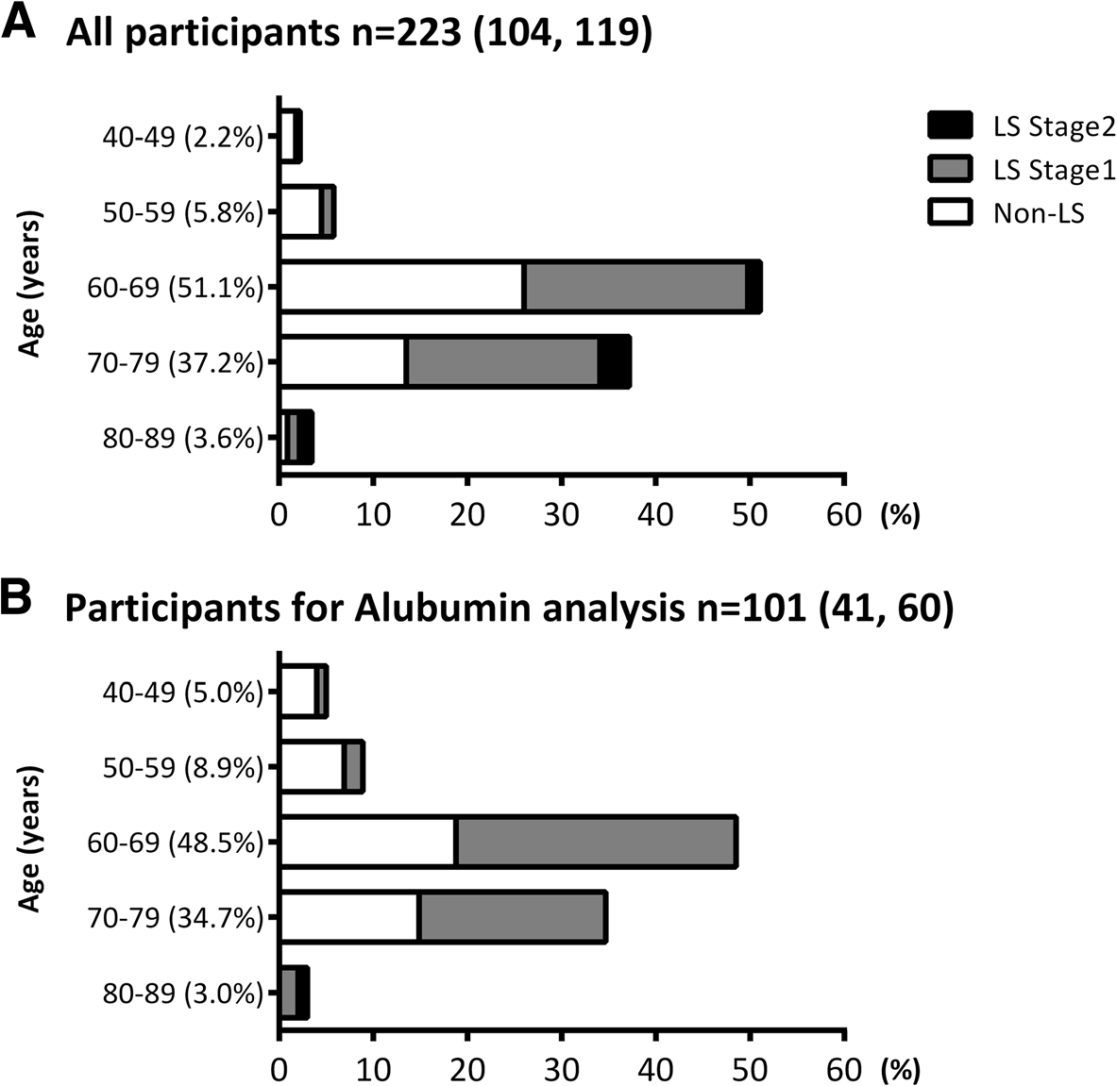
Age distribution of the locomotive syndrome status. **a**) all participants, **b**) albumin analysis participants. □: Non-LS participant, ■: LS Stage 1 participant, ■: LS Stage 2 participant

The level of serum albumin is shown in Fig. 2. In the current study, the blood samples of 101 of 223 participants (41 men, 60 women) were analyzed for serum albumin level, with an LS prevalence of 55.4% (56 of 101, 24 men, 32 women). This LS prevalence was approximately equivalent to the cohort of our study (119 of 223 [53.3%]; 55 men, 64 women) (Fig. 3). Serum albumin level was significantly lower in the LS group than in the non-LS group (non-LS vs LS: 4.40 ± 0.27 vs. 4.26 ± 0.22%, *p* = 0.0070). Moreover, 29 of 56 (51.8%) and 12 of 45 (26.7%) participants had low albumin levels in the LS and non-LS groups, respectively.

Moreover, the ROC analysis indicated that the cutoff point was 4.3 g/dL for albumin (AUC; 0.69, sensitivity; 58.8%, specificity; 79.9%), and high level of albumin significantly lowered the LS risk (*p* = 0.0445) with an OR of 0.148 (OR_95%CI_ = 0.023–0.954).

Additionally, significant correlations were observed between albumin level and % Fat or albumin and total skeletal muscle mass (% Fat, r = 0.2149; skeletal muscle mass, r = − 0.2196, *p* <  0.05) (Table 3). Furthermore, there were significant differences in albumin level between LS and non-LS when the two-step test were used (*p* = 0.0178) (Table 4).

## Discussion

This study aimed to determine the association between the components of LS and blood parameters in the Japanese middle-aged and elderly people. In our study, 119 of 223 subjects (53.3%) fulfilled the diagnostic criteria for LS. The participants with LS had lower relative knee extension strength (weight bearing index of knee extension) and slower walking speeds than the non-LS participants, suggesting that the onset of mobility decline is diagnosed in accordance with previous cross-sectional studies [1, 5, 18]. The present study demonstrated, for the first time to the best of our knowledge, that higher levels of HbA1c and lower serum albumin were associated with the prevalence of LS in the Japanese middle-aged and elderly people. Furthermore, DHEA-S levels may be a useful screening tool for LS in men.

The main findings of this study were that higher HbA1c levels were associated with a higher prevalence of LS, and that the OR for participants with levels above the threshold value was 2.62 in the LS group. Our data suggest that the participants with HbA1c level ≥ 5.7% had a higher risk of LS than those with HbA1c level < 5.7% among the Japanese middle-aged and elderly individuals. HbA1c, one of the diagnostic criteria for diabetes, is the most widely used indicator of glucose control, and can be used to assess overall metabolic control over the preceding 6–8 weeks [19]. The American Diabetes Association and the World Health Organization recently proposed the use of HbA1c to diagnose diabetes at threshold levels of 6.5% (48 mmoL/moL) and to identify individuals at high risk of developing type 2 diabetes based on a cutoff value of 5.7% (39 mmoL/moL) [20, 21]. In contrast, a meta-analysis by Boulé et al. [22] demonstrated that reductions in the HbA1c levels following an aerobic training program in diabetic patients were better predicted by exercise intensity than by exercise volume. Moreover, our data showed that the HbA1c level negatively correlated with the maximal 10-m walking speed and positively correlated with the BMI; therefore, HbA1c levels seem to be higher in individuals who do not regularly perform exercise or who maintain reduced physical activity. Although the relationship between LS and other diseases, such as diabetes, cardiovascular disease, and other age-related diseases, is unknown, we nonetheless consider that higher HbA1c levels may be one of the characteristics of increased risk of LS among Japanese middle-aged and elderly people.

In the present study, the prevalence of LS was higher in men with lower DHEA-S levels (cutoff: 88 μg/dL). Indeed, although we could not show a linear relationship between DHEA-S level and physical function (muscle strength and gait speed, data not shown), LS men subjects exhibited lower muscle strength in knee extension in accordance with the results of a previous study by Valenti et al. [12]. DHEA-S is an endogenously produced sex steroid that has been hypothesized to have anti-aging effects [9]. Circulating DHEA-S levels decline significantly with age, resulting in mean levels at the age of 65 years being less than one fifth of those at the age of 20 years, according to cross-sectional [11] and longitudinal [10] studies. Moreover, several studies have demonstrated that low levels of DHEA-S are associated with cardiovascular disease [23] and muscle weakness [12] in older men. In older women, either low or high levels of DHEA-S compared to reference levels, confer a greater risk of death (cardiovascular or cancer mortality) than do intermediate levels [24]. Considering that several longitudinal studies have confirmed that 30% of individuals older than 65 years do not experience a decline in DHEA-S levels [25–27]; therefore, the clinical significance of DHEA-S remains unclear. Moreover, there were no associations between DHEA-S levels and LS in women. This may be due to sex differences in the DHEA-S levels (women tend to have lower levels of DHEA-S), suggesting that the physiological significance of DHEA-S in women may be less. Therefore, more research is needed to determine whether targeted DHEA-S supplementation would provide clinical benefit for preventing LS in men. Nonetheless, our data suggest that low DHEA-S levels may be a useful tool for identifying increased risk of LS in middle-aged and older men.

Interestingly, we also found that lower levels of serum albumin (< 4.3 g/dL) were significant risk factors for LS. In general, serum albumin is a marker of nutritional condition [28], acts as an antioxidant [29], and is a plasma volume expander [30]. Although, we have no data to support the relationship between reduced albumin level and LS, lower levels of serum albumin may be associated with a decline in the muscle mass. LS is partly caused by weakness and loss of skeletal muscle with aging, so called sarcopenia, which results in drastically reduced qualities of life, and leads to an increased risk for the development of insulin resistance and various diseases [31, 32]. Our data indicated that the albumin level was negatively correlated with the total skeletal muscle mass measured via bioelectrical impedance analysis. Consequently, a lower level of albumin may be associated with decline in the skeletal muscle mass, thus appearing to be related to increased risk of LS. Although it was sufficient to detect statistically significant changes in the variables, the sample size for the serum albumin analysis was relatively small (*n* = 101 [41 men, 60 women]) compared to the other blood parameters (*n* = 223) in the present study. Therefore, additional studies are required to clarify the significance of serum albumin levels in predicting an increased risk for LS among Japanese middle-aged and elderly individuals.

### Limitations

This study had several limitations. First, the sample size of 223 was small, particularly, the sample size that underwent albumin analysis (101 of 223). Second, we did not control the diet before the experiment. Although we had taken the blood samples after at least a 3-h fast, we did not analyze the influence of diet intake on the blood parameters. Third, we could not examine the causal relationship between HbA1c, DHEA-S (for men), and albumin levels and LS because of the cross-sectional study design. Therefore, a future follow-up study is necessary to clarify the causal relationships among these factors. Fourth, our participants were limited to relatively healthy individuals, especially elderly people; therefore, our findings may not be extrapolated to the entire Japanese population. Finally, as shown in Table 4, there were significant differences in HbA1c and Albumin levels between LS and non-LS for the two functional tests (stand-up test and/or two-step test), while not for GLFS-25. That is, given that the criteria of LS would differ by measurement tools (functional tests or GLFS-25), future studies should be focused on the optimized criteria of the LS to predict an increased risk for LS with higher accuracy. Despite these limitations, our data first demonstrated that higher levels of HbA1c and lower levels of serum albumin were associated with a higher prevalence of LS among the Japanese middle-aged and elderly individuals.

## Conclusions

Our data suggested that higher HbA1c and lower albumin levels were associated with a higher prevalence of LS among the Japanese middle-aged and elderly people. Furthermore, low DHEA-S levels may be a useful screening tool for LS in men.

## Abbreviations

ALP: Alkaline phosphatase
ALT: Alanine amino transferase
AST: Aspartate amino transferase
BMI: Body mass index
CI: Confidence interval
DHEA-S: Dehydroepiandrosterone-sulfate
GH: Growth hormone
GLFS: Geriatric locomotive function scale
Hb: Hemoglobin
HbA1c: Glycated hemoglobin
Hct: Hematocrit
HDL-C: High-density lipoprotein cholesterol.
IGF-1: Insulin-like growth factor-1
IQR: Interquartile range
JOA: Japanese Orthopedic Association
LAP: Leucine aminopeptidase
LDL-C: Low-density lipoprotein cholesterol
LS: Locomotive syndrome
NGSP: National Glycohemoglobin Standardization Program
OR: Odds ratio
ROC: Receiver operating characteristics
SD: Standard deviation
TG: Triglyceride
TP: Total protein.
WBC: White blood cell
γ-GTP: γ-glutamyl transpeptidase
